# Whole-genome bisulfite sequencing of cell-free DNA identifies signature associated with metastatic breast cancer

**DOI:** 10.1186/s13148-015-0135-8

**Published:** 2015-09-16

**Authors:** Christophe Legendre, Gerald C. Gooden, Kyle Johnson, Rae Anne Martinez, Winnie S. Liang, Bodour Salhia

**Affiliations:** Integrated Cancer Genomics Division, Translational Genomics Research Institute, 445 N Fifth Street, Phoenix, AZ USA

## Abstract

**Background:**

A number of clinico-pathological criteria and molecular profiles have been used to stratify patients into high- and low-risk groups. Currently, there are still no effective methods to determine which patients harbor micrometastatic disease after standard breast cancer therapy and who will eventually develop local or distant recurrence. The purpose of our study was to identify circulating DNA methylation changes that can be used for prediction of metastatic breast cancer (MBC).

**Results:**

Differential methylation analysis revealed ~5.0 × 10^6^ differentially methylated CpG loci in MBC compared with healthy individuals (H) or disease-free survivors (DFS). In contrast, there was a strong degree of similarity between H and DFS. Overall, MBC demonstrated global hypomethylation and focal CpG island (CPGI) hypermethylation. Data analysis identified 21 novel hotspots, within CpG islands, that differed most dramatically in MBC compared with H or DFS.

**Conclusions:**

This unbiased analysis of cell-free (cf) DNA identified 21 DNA hypermethylation hotspots associated with MBC and demonstrated the ability to distinguish tumor-specific changes from normal-derived signals at the whole-genome level. This signature is a potential blood-based biomarker that could be advantageous at the time of surgery and/or after the completion of chemotherapy to indicate patients with micrometastatic disease who are at a high risk of recurrence and who could benefit from additional therapy.

**Electronic supplementary material:**

The online version of this article (doi:10.1186/s13148-015-0135-8) contains supplementary material, which is available to authorized users.

## Background

A number of clinico-pathological criteria have been established as breast cancer prognostic markers to determine risk of recurrence and stratify patients into high- and low-risk groups. The likelihood of distant metastasis increases with tumor size, the presence and number of lymph-node involvement (≥4 nodes have a higher recurrence risk), lack of estrogen receptor (ER) expression, over-expression of Her2, a high proliferative index, lymphovascular invasion, and loss of histopathological differentiation [1].

Molecular profiles have improved our ability to determine the need of chemotherapy for those individuals who are deemed high-risk. The most widely used multigene classifiers include the 21-gene Oncotype Dx signature (Genomic Health, USA), the 70-gene MammaPrint signature (Agendia, Netherlands), the 76-gene Rotterdam signature, and the PAM50 intrinsic classifier (NanoString, USA) [2]. Despite the huge quantity of information gleaned from these gene signatures, none can precisely predict the clinical course of an individual and rely on the presence of tissue at a single time point. Therefore, they are not able to monitor a patient’s risk status after completion of therapy due to residual disease. Even with the clinico-pathological features, there are patients deemed high-risk who do very well with standard therapy and never experience a recurrence and patients with low-risk profiles who still die of breast cancer. There also remains a risk of recurrence even after the most effective chemotherapy agents are administered to high-risk patients. We report a 21-gene DNA hypermethylation signature, detectable in the circulation of MBC patients, which maybe useful in the pre-macrometastatic setting to indicate patients at a high risk of recurrence.

## Results

### Clinical characteristics of samples

We characterized the plasma methylome of MBC by paired-end whole-genome bisulfite sequencing (WGBS) to identify differentially methylated regions that were uniquely found in circulating cfDNA of a pool of 40 MBC when compared with a pool of 40 H and a pool of 40 DFS. MBC samples represented metastasis to usual sites including bone (*n* = 23), liver (*n* = 12), brain (*n* = 3), lung (*n* = 17), and soft tissue (*n* = 6) (Additional file 1: Figure S1A). All but five samples had involvement of more than one site. For the DFS cohort, the average years disease-free equals 9, with a range of 3-27 years (Additional file 1: Figure S1B). The groups were relatively matched for age at diagnosis and race (Additional file 1: Figure S1D-E). The median age for H, DFS, and MBC was 48, 42, and 42, respectively (Additional file 1: Figure S1D). Furthermore, the DFS and MBC groups showed comparable hormone-receptor and Her2-receptor status and prior therapy regimens (Additional file 2: Table S1).

### Summary of WGBS statistics

For quality control assurances, we confirmed that cfDNA-fragment sizes were near equal between samples pre- and post-fragmentation, and the DNA library yields and percent-alignment rates were nearly equal for the three sample pools (Additional file 3: Figure S2). A total of approximately 504, 625, and 948 million reads were obtained for H, DFS, and MBC, respectively, using ten lanes of sequencing on an Illumina HiSeq 2500 (Additional file 4: Table S2). Among these reads, a mean of 64.3 % of reads were nonduplicated. A final read count of ~227 (H), ~295 (DFS), and ~518 (MBC) million reads were used for downstream analyses. The average depth of coverage after deduplication was 7.4 (H), 9.6 (DFS), and 16.9 (MBC). The number of CpG sequenced was 28,162,972. Of these CpGs, 61.9, 74.8, and 85.7 % were included in further analysis in H, DFS, and MBC, respectively. The increased coverage in MBC was not due to global copy number alterations as captured by SVDetect (data not shown).

### WGBS demonstrated global hypomethylation and focal hypermethylation in cfDNA of MBC compared with H and DFS, which had a high degree of similarity

**Fig. 1:**
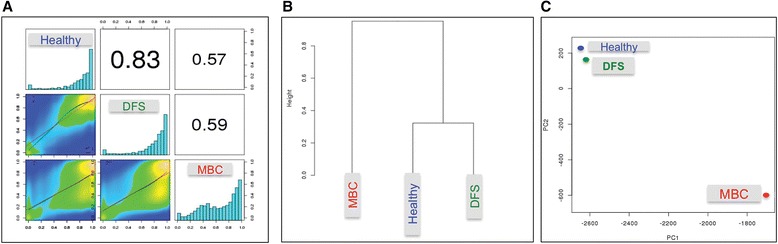
WGBS reveals that MBC methylation profiles differ from DFS and H, which are similar. **a** Heat scatterplots show % methylation values for pair-wise comparisons of three study groups. Numbers on the *upper right corner* denote Pearson correlation coefficients. The histograms on the diagonal are frequency of % methylation per cytosine for each pool. MBC demonstrates a shift to the left compared to the DFS and H, indicating genome-wide hypomethylation. **b** Hierarchical clustering of methylation profiles for each pool using Pearson’s correlation distance and Ward’s clustering method. **c** Principal Component Analysis of the methylation profiles of each cfDNA pool, showing PC1 and PC2 for each sample. Samples closer to each other in clustering or principal component space are similar in their methylation profiles

To assess the similarity of each sample group to the others, we used methylKit (25) to compute pair-wise Pearson correlation coefficients, hierarchical clustering (Ward’s method, correlation distance metric), and Principal Component Analysis (PCA) on % CpG methylation profiles. These analyses demonstrated that the H cohort closely resembled DFS, evidenced by Pearson correlation coefficient (0.83) and close proximity by hierarchical clustering and PCA (Fig. 1). However, MBC varied dramatically from H and DFS according to each analysis type, where the Pearson correlation coefficients were 0.57 and 0.59 and showed a large degree of separation by clustering and PCA. The percent methylation values per base for each sample group demonstrated that the majority of loci in DFS and H were methylated (major peak close to 1), whereas MBC had a significant proportion of loci shifted to the left indicating low methylation states and hypomethylation compared to H and DFS (Fig. 1a). To rule out a chromosomal bias, we performed this analysis for each chromosome (excluding X and Y) and confirmed a similar trend (Additional file 5: Figure S3).

### Identification of 21 CpG island hypermethylated hotspots in circulation of MBC

**Fig. 2:**
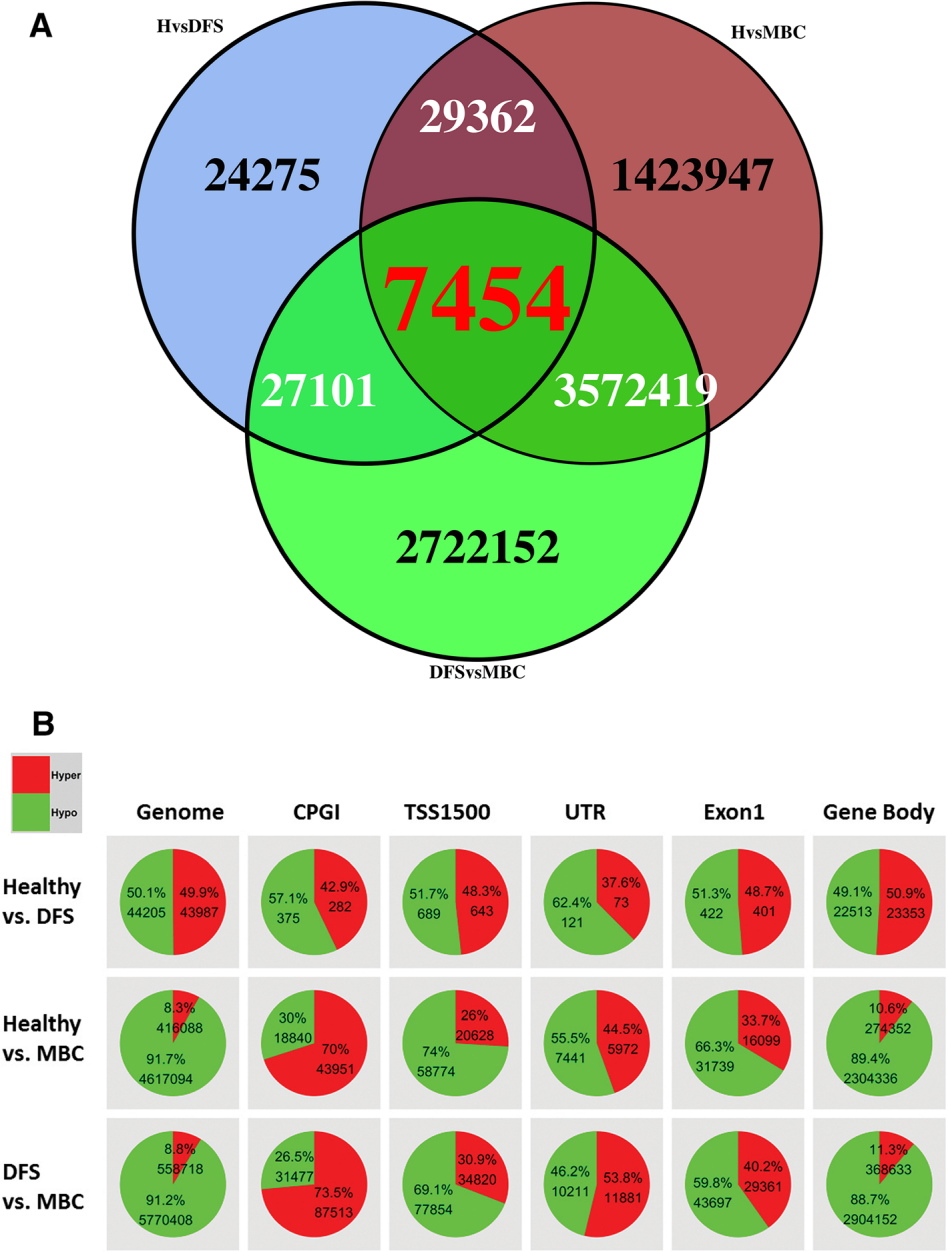
**a** Venn diagram showing the overlap of DML lists as generated by WGBS for H, DFS, and MBC sample comparisons. **b** Three pair-wise comparisons assessing cfDNA differential methylation between H, DFS, and MBC. *Pie charts* show percentages of differentially hyper- or hypomethylated CpG loci genome-wide and within the displayed genomic contexts. Greater than 90 % of CpG loci are hypomethylated genome-wide in MBC compared with Healthy or DFS. The majority of hypermethylated loci in MBC occur within CpG islands. The number of DML and the percentages are shown within each *pie chart*

We also used methylKit to perform pair-wise differential methylation analysis at a single base-pair level. The number of differentially methylated loci (DML) between H and DFS was relatively small (*n* = 88,192), again indicating the similarity between the groups. In contrast, ~6.3 × 10^6^ DML were detected between MBC and DFS and ~5.0 × 10^6^ DML detected between MBC and H (Fig. 2a). A Venn diagram (Fig. 2a) showing the overlap of DML from each comparison demonstrates a high degree of overlap when MBC is compared to either H or DFS. However, very little overlap exists with the H vs. DFS DML list when compared to the DML list generated in the two MBC comparisons. Greater than 90% of DML were hypomethylated in MBC compared with either H or DFS, indicating genome-wide global hypomethylation in the plasma of MBC (Fig. 2b). To discern the biological impact of differentially methylated loci, each event was put into a genomic context: CpG island, TSS1500, UTR, Exon 1, and Gene Body (Fig. 2b). Approximately 9 % of DML were hypermethylated in MBC compared to either H or DFS. The greatest number of hypermethylated DML occurred in CPGIs (~70 %). There was also significant (*P* value <0.05) hypermethylation occurring in UTRs (~50 %), Exon 1 (~35 %), and TSS1500 (~30 %). Hypermethylation occurred least frequently in gene bodies (~11 %), which were predominately hypomethylated.

**Fig. 3:**
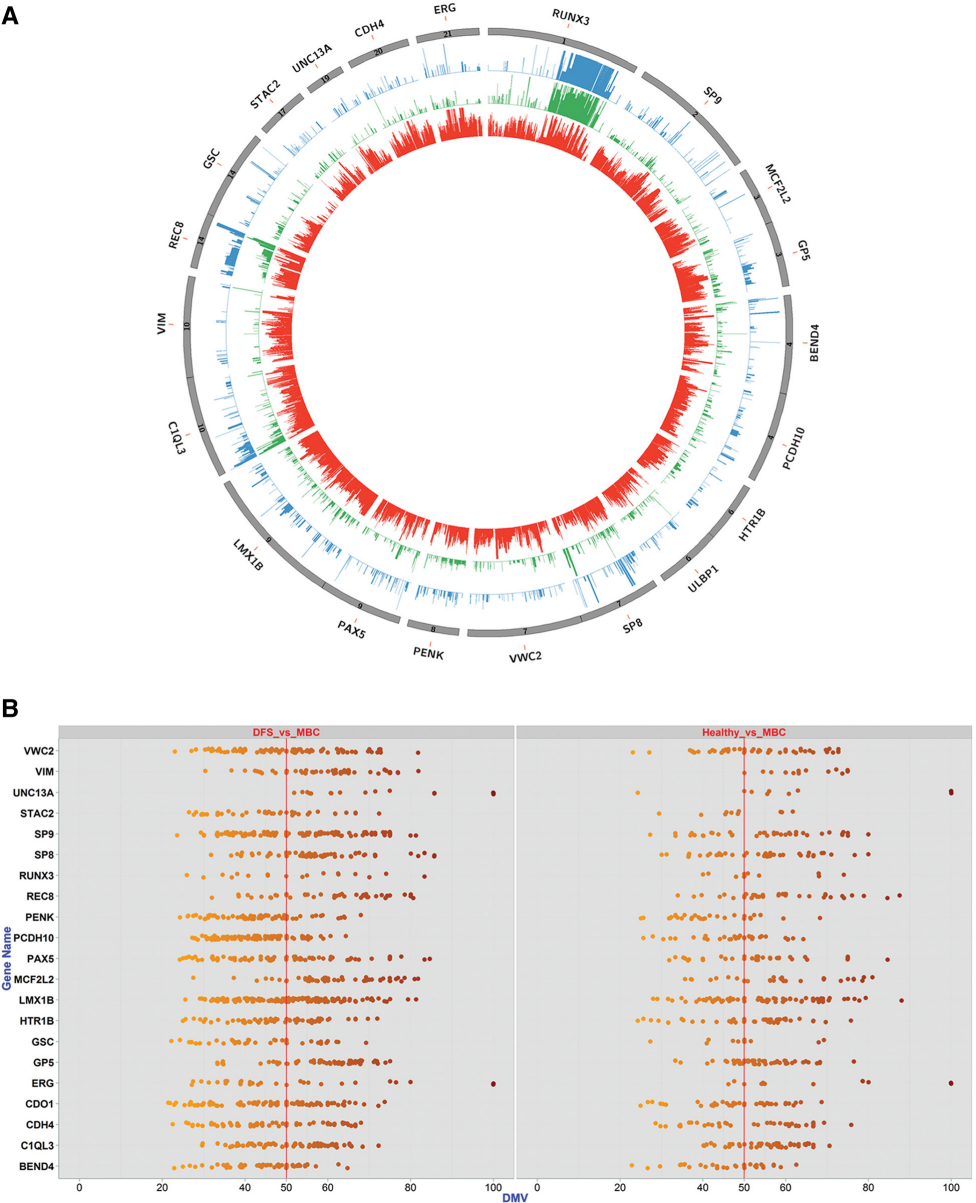
**a** Circos plot graphing methylation state for each locus in the CpG island of 21 target genes. The hotspot region exists within each island. The *inner circle* (*red*) is MBC, *middle circle* is DFS (*green*), and *outer circle* is H (*blue*). Hypermethylation is evident in MBC for the target genes. **b** Vertical scatter plot showing all DML within target CPGIs for MBC versus DFS and H, respectively. Each *point* represents a CpG locus. *Points* plotted on the *x*-axis display the DMVs

To mine the data for potential biomarkers of MBC, we focused on hypermethylated loci specifically in CPGIs because they tend to be focal in nature and were identified as the regions that differed most dramatically from normal or disease-free patterns. We specifically selected regions with eight or more hypermethylated loci with differential methylation values (DMVs) ≥50. With these criteria, we identified 21 CPGI hotspots, which we refer to as CpG4C™, within the following genes: BEND4, CDH4, C1QL3, ERG, GP5, GSC, HTR1B, LMX1B, MCF2L2, PAX5, PCDH10, PENK, REC8, RUNX3, SP8, SP9, STAC2, ULBP1, UNC13A, VIM, VWC2 (Fig. 3).

### Validation of WGBS using targeted bisulfite amplicon sequencing with MiSeq

**Fig. 4:**
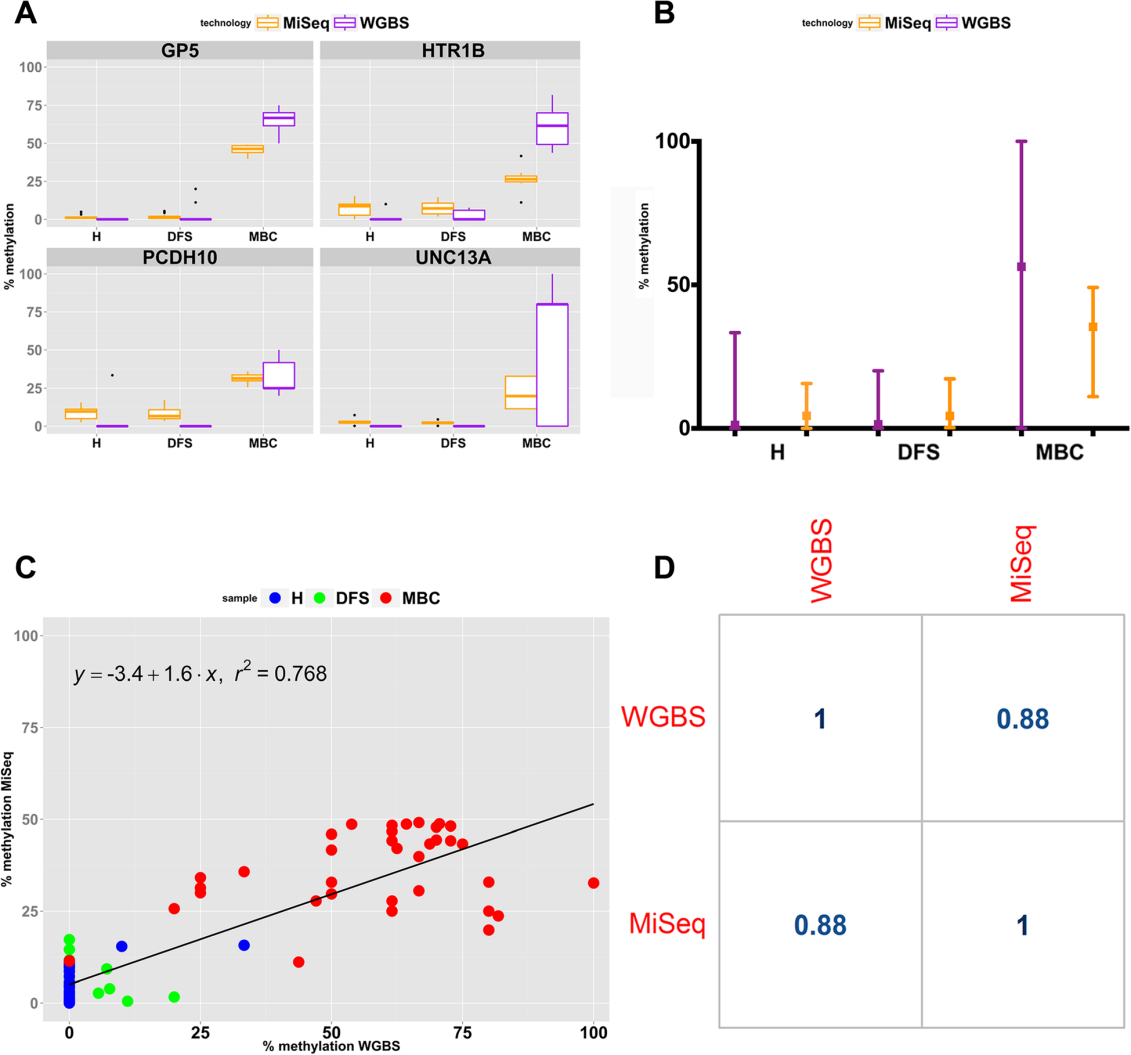
Comparison of WGBS to MiSeq (targeted amplicon sequencing). **a** *Box plots* representing percent methylation for DMLs in GP5, HTR1B, PCDH10, and UNC13A as called by both technologies. **b** Mean-Whisker plots displaying average methylation state of all amplicons assayed by MiSeq and WGBS. **c** *Scatter plot* of percent methylation value for the 36 CpGs assayed in H, DFS, and MBC by both MiSeq and WGBS. The correlation is reported as *R* ^2^ = 0.768. **d** Pearson correlation coefficient for WGBS versus MiSeq for 36 CpGs assayed by targeted amplicon sequencing

We used bisulfite amplicon sequencing on Illumina’s MiSeq platform for technical validation of WGBS on an independent extraction of plasma from each group. This nascent, deep-sequencing strategy allows for sensitive detection of DNA methylation in low-input samples such as plasma. Due to sample limitations, we were not able to technically validate all 21 CpG hotspots, so we randomly selected 4/21 genes for technical validation using MiSeq. We selected GP5, UNC13A, PCDH10, and HTR1B genes and designed bisulfite PCR primers within the region of interest. Each amplicon detected between 6–18 CpG loci (Additional file 6: Figure S4A-D). Targeted bisulfite amplicon sequencing on the MiSeq platform showed very good concordance with WGBS and demonstrated statistically significant (*P* value <0.05) increased methylation in MBC compared with H and DFS in GP5, PCDH10, HRR1B, and UNC13A (Fig. 4a, b, Additional file 6: Figure S4A-D). The MiSeq data also maintained that H and DFS are virtually unmethylated within these amplicons (Fig. 4a, b and Additional file 6: Figure S4A-D). All comparisons between MBC and H or DFS were statistically significant (*P* value <0.05) by Fisher’s Exact Test and ANOVA, while surviving multiple test correction (*q* value ≤0.5). To further assess the degree of correlation between MiSeq and WGBS data for the amplicons containing the 36 CpG assayed, we performed a scatter plot analysis and a Pearson correlation analysis to compare the 36 loci, for all groups, between the two technologies. This analysis demonstrated a high degree of correlation between MiSeq and WGBS (*R*^2^ = 0.768 and Pearson Correlation = 0.88) (Fig. 4c, d). All loci in H and DFS (green and blue dots, respectively) clustered to very low methylation states to the lower left of the graph and CpG loci in MBC (red dots) mostly scattered to the upper right (Fig. 4c). A summary of the percent methylation values for each technology across the groups is shown in Additional file 7: Table S3.

**Fig. 5:**
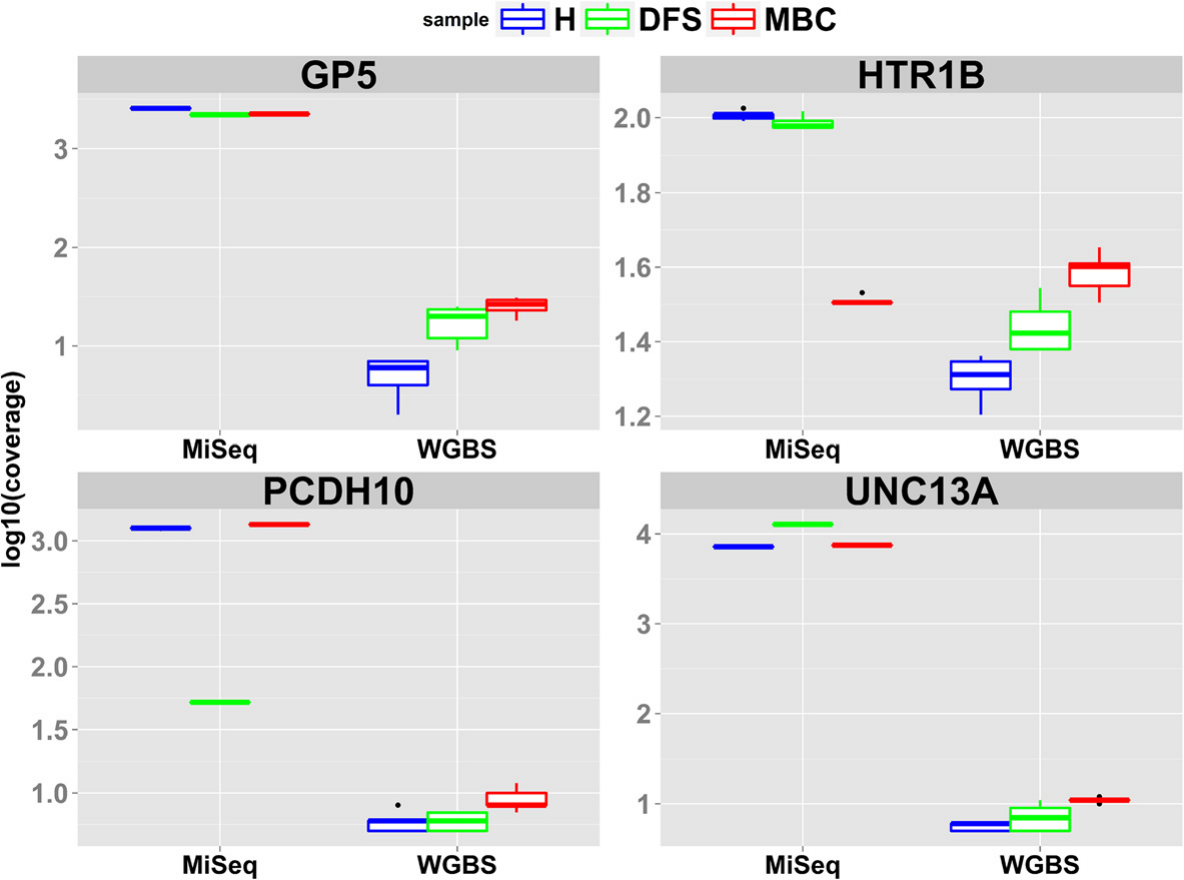
Read coverage in DMLs of interest. *Box plots* show the depth of sequencing as determined by WGBS and MiSeq for 36 DMLs specific to GP5, HTR1B, PCDH10, and UNC13A in all pools of H (*blue*), DFS (*green*), and MBC (*red*). Coverage is shown as log10

To demonstrate the expected higher coverage of MiSeq with WGBS, we calculated the mean depth of coverage for each CpG locus, within each amplicon, for each group (Fig. 5). The overall average depth of coverage for the 36 CpG loci in H, DFS, and MBC by WGBS was 10, 9.4, and 11. The average number of reads for H, DFS, and MBC by MiSeq was 3012, 2583, and 2516, respectively.

### Gene ontology implications for CpG4C™

In order to demonstrate the association of the 21 gene panel to biological processes we performed the Core Analysis in Ingenuity® Pathway Analysis (IPA®). The top disease implication was Cancer showing involvement of 17/21 genes (Additional file 8: Table S4A). The Top Molecular and Cellular Function was Cell-Cell Signaling and Interaction (Additional file 8: Table S4A). Within the Cancer disease process, 17 genes were associated with Digestive System Cancer (Additional file 8: Table S4B). VIM and CDH4 were implicated in invasive cancer (Additional file 8: Table S4B).

## Discussion

Cancer metastases arise from disseminated cells of the primary tumor mass before treatment and/or from minimal residual disease (MRD) persisting after therapy (collectively known as micrometastatic residual disease) [3]. Currently, there are still no effective methods to determine which patients harbor micrometastatic disease after standard breast cancer therapy and who will eventually develop local or distant recurrence. It would be advantageous to determine the subset of patients who harbor micrometastatic cells and develop trials that would evaluate the use of additional therapy for eventual prevention of metastasis. There is likely a predictive clinical window of opportunity to detect microscopic disease in the early disease setting before micrometastases lead to incurable macrometastases years after initial diagnosis.

This study represents one of the first whole-genome studies describing the plasma methylome and the first unbiased study reporting the circulating methylome of MBC, resulting in the identification of a 21-gene hotspot methylation panel that can potentially be used for prediction of metastasis in the pre-macrometastatic setting. Also novel to this study is the comparison of the plasma methylome of MBC to that of both H and DFS, making the DML hotspots highly unique to patients with clinical evidence of MBC. While other studies have reported the detection of tumor-associated DNA methylation changes in cfDNA, targets were usually selected a priori from tissue microarray data and measured using targeted approaches and not directly associated with MBC [4–9]. Furthermore, we demonstrate that genome-wide DNA methylation profiles of DFS resemble plasma methylomes from healthy individuals. This suggests that methylation patterns in cfDNA can be used to discriminate a true signal from normal-derived, background noise; the patterns may be used to detect the presence of micrometastatic residual disease after therapy. Additionally, we show that the circulating methylomic landscape of MBC is congruent with our knowledge of a cancer cell’s DNA methylation patterns, characterized by global genome-wide hypomethylation and focal hypermethylation, found most frequently in CPGIs. Accordingly, the data demonstrate, as one would expect, that the hypermethylated regions detected are regions that are generally unmethylated in the genome. Previously, Chan et al. also observed hypomethylation in cfDNA of a variety of cancers; albeit, the study did not discuss hypermethylation or MBC and did not report on specific genic events [10].

Of the 21 genes, hypermethylation of RUNX3, PENK, PAX5, and PCDH10 have been implicated in breast cancer [11–13]. We have previously reported the association of PENK hypermethylation in breast cancer metastasis to the brain [14]. RUNX3, GSC, CDH4, BEND4, PENK, VIM, and PCDH10 have been previously associated to invasion and metastasis [14–20]. DNA methylation alterations in UNC13A, SP9, GP5, C1QL3, SP8, and VWC2 have not been previously reported in cancer.

A potential limitation in our study lies in the pooling approach we used to conduct our analysis. In the absence of individual sample analysis, and given the dynamic range of circulating DNA [21], one cannot be certain that a few samples are not overshadowing the other samples, thus reducing the complexity of the pool. However, the expense of such a large-scale analysis such as WGBS remains prohibitive. In addition, other studies have reported that bisulfite-based epityping on pooled genomic DNA provided accurate estimates of average group DNA methylation [22, 23]. Still, the importance of individual sample and alternate cohort validation are critical to future development of this potential biomarker. In this study, we have demonstrated cross-platform validation using targeted bisulfite sequencing on MiSeq; this validated the results of WGBS for our hotspots selected within GP5, HTR1B, PCDH10, and UNC13A. More extensive validation could not be completed due to sample limitations and study scope. However, we are currently working on determining the sensitivity and specificity of CpG4C in additional samples that we are acquiring.

Various types of DNA alterations have been reported in cfDNA including point mutations, microsatellite instabilities, loss of heterozygosity, and DNA hypermethylation [24, 25]. The essentiality of proper DNA methylation maintenance is highlighted in cancer, where normal patterns are lost. Aberrant DNA methylation is among the earliest and most chemically stable molecular alterations in cancer, making it a potentially useful biomarker for early detection or risk prediction [8, 26]. The high degree of detection sensitivity of aberrantly methylated loci is afforded by the frequency of the occurrence (for example, compared to somatic mutations) and because bisulfite modification provides detection of hypermethylated targets in large excess of unmethylated ones (1:1000) [26]. Still, important issues like temporal stability of DNA methylation in biological fluids need to be better assessed. A study by Byun et al. demonstrated that that degree of short-term DNA methylation stability is marker dependent and associated with sequence characteristics and methylation levels [27]. Such factors will be of the utmost importance when designing and conducting future clinical tests using circulating epigenetic markers.

Early reports suggesting that the simple presence or absence of cfDNA itself, or its concentration was diagnostic [8], have been scrutinized; high levels of cfDNA are not specific to neoplastic lesions and are also observed in several other pathologies, including pro-inflammatory and neurological disorders [24]. In addition, cfDNA has also been found in healthy individuals in the same concentration range of some cancer patients. Our lab has corroborated this finding by demonstrating a fairly equal distribution of DNA yields in plasma from H, DFS, and MBC patients (Additional file 1: Figure S1C). This argues that the presence of tumor-specific alterations is the best criterion to assess the tumoral origin of cfDNA.

## Conclusions

In summary, this unbiased analysis of cfDNA identified 21 DNA hypermethylation hotspots associated with MBC, and demonstrated the ability to distinguish tumor-specific changes from normal-derived signals at the whole-genome level. We anticipate that a DNA hypermethylation signature, involving rationally selected CpG hotspots detectable in circulation, can be used to indicate micrometastatic disease in the pre-macrometastatic setting and predict patients at a high-risk of recurrence who could benefit from additional therapy. Future studies, involving targeted bisulfite amplicon sequencing on individual samples, and in samples from early stage breast cancer, will further validate the predictive power of this signature and may further help define its association to varying breast cancer subtypes.

## Methods

### Sample acquisition and DNA extraction

We obtained 120 retrospectively collected plasma samples from the Komen Tissue Bank (KTB), IU Simon Cancer Center representing 3 cohorts of 40 individuals: cohort 1 is MBC to various organs; cohort 2 is DFS (range: 3–27 years, average 9 years DFS); cohort 3 is H with no history of cancer. Samples were obtained under informed consent following Komen Tissue Bank Institutional Review Board approval. Plasma collection and processing is critical to the reproducibility of tests involving cfDNA. The KTB uses a highly standardized and meticulous protocol for processing plasma to ensure separation from blood and subsequent storage in a highly time efficient manner. Details on KTB’s plasma collection SOP can be found on their website (http://komentissuebank.iu.edu/researchers/standard-operating-procedures/). A plasma pool for each cohort was created by mixing 50 μl of a pre-aliquoted plasma sample per individual, followed by extraction of cfDNA from 1 ml of each pool using the QIAamp DNA Micro Kit (Qiagen) according to the manufacturer’s protocol, with the exception that we used 1 μg of carrier RNA. DNA yields from four independent 1-ml extractions of each pool were highly consistent. The manufacturer’s protocol for “Isolation of Genomic DNA from Small Volumes of Blood” was followed, with the exception that reagents were scaled up proportionally, and the sample was serially extracted on the column to accommodate the increased volume. DNA was eluted in AE Buffer (Qiagen) and quantified using the Qubit dsDNA High Sensitivity fluorometric assay (Invitrogen).

### DNA methylation analysis by whole-genome bisulfite sequencing

Directional bisulfite-converted libraries for paired-end sequencing were prepared using the Ovation Ultralow Methyl-Seq Library System (NuGen). The manufacturer’s suggested protocol was followed. Briefly, this entailed fragmentation, end repair, adapter ligation, final repair, bisulfite conversion, and PCR amplification. We used 27, 14, and 33 ng of DNA for H, DFS, and MBC, respectively, in 50 μl T low E buffer, which was fragmented to an average size of 200 bp using the Covaris S2 system (Additional file 3: Figure S2A). Bisulfite conversion was performed using the EpiTect Fast DNA Bisulfite Kit (Qiagen) as per manufacturer’s instructions. Post-library QC was performed with BioAnalyzer DNA 1000 chips (Agilent) and the Qubit dsDNA High Sensitivity fluorometric assay (Invitrogen). An equimolar pool of the prepared libraries was created at a concentration of 5 nM. The sample was subsequently diluted and clustered on the Illumina cBot using TruSeq Paired End Cluster Kit v.3 chemistry. Paired-end sequencing was performed on the Illumina HiSeq 2500 platform using TruSeq SBS v3 kits for a total read length of 200 bp.

### Targeted bisulfite amplicon sequencing

Targeted bisulfite amplicon sequencing was performed on the MiSeq (Illumina) using an independent replicate of the three plasma pools for validation of CpG island hotspots for GP5, HTR1B, PCDH10, UNC13A. Bisulfite Primer Seeker 12S (Zymo Research) was used to create primer-pairs specific for bisulfite-converted DNA, which produced PCR amplicons ranging in size from 109–235 base pairs. The bisulfite conversion was accomplished using EZ DNA Methylation-Gold Kit (Zymo Research) according to the manufacturer’s standard protocol. Forty cycle PCR reactions were carried out with the Zymo Taq (Zymo Research) kit and the manufacturer’s recommended conditions using 2 μl of converted DNA template per 30 μl reaction. Reactions were purified using NucleoSpin columns (Macherey-Nagel) as per the manufacturer’s suggested protocol. Purified reaction products were run out on a 2 % agarose gel for visual inspection and quantified using the Qubit dsDNA High Sensitivity fluorometric assay (Invitrogen).

A 266-ng equimolar mix of the four amplicons was used as input for sequencing library preparation using the Kapa Hyper Prep Kit (Kapa Biosystems). TruSeq DNA LT adapters (Illumina) were used for indexing. No post-ligation amplification was performed. Quantitative-PCR library quantification was carried out using the Kapa Library Quantification Kit (Kapa Biosystems).

Equimolar library pools were created and diluted to 15 pM for denaturation. PhiX Control v3 (Illumina) was spiked in at a 5.0 % final concentration, and subsequent cluster generation/sequencing was performed on the MiSeq using MiSeq Reagent Nano Kits (Illumina). Five hundred cycles of 2 × 250 paired-end sequencing generated over 820,000 reads.

### Data processing and analysis

Bisulfite-modified DNA reads from WGBS and MiSeq were aligned to the bowtie2-indexed reference genome GRCh37-62 using Bismark tool version 0.12.7 [28]. Bismark relies on two external tools, bowtie (http://bowtie-bio.sourceforge.net/index.shtml) and Samtools (http://www.htslib.org). We respectively used bowtie2 version 2.0.0-beta6, and Samtools version 0.1.19. Bismark was used as suggested except for the bowtie2’s parameter *N* (number of mismatches in a seed alignment during multispeed alignment) where the value of 1 was used for increased sensitivity. Next, PCR duplicates were removed for WGBS using default parameters. Methylation calling was also processed using a Bismark module called “Methylation Extractor,” which was used according to the author’s specifications. Base-pair level differential methylation analysis was implemented using the R package methylKit 0.9.2 [29]. Bismark’s sam file output was used as input to methylKit and data imported using the embedded function “read.bismark”. The minimum read coverage to call a methylation status for a base was set to 5, and the minimum phred quality score to call a methylation was set to 20. The read.context option was set to “CpG”. Other options to the read.bismark function were set to default values. The following pair-wise comparisons were performed in methylKit using the Fisher Exact Test: H versus DFS, H versus MBC, and DFS versus MBC for both WGBS and MiSeq datasets. Before calling differential methylation, each comparison was methylKit-reorganized, united, and then underwent differential methylation analysis using methylKit functions. With a minimum of five reads in each group, a differential methylation value (DMV) of 20 (in percent scale) and *P* values <0.05 were considered DML. For WGBS and MiSeq, chromosome X and Y reads were removed. MethylKit DML calls were annotated according to genomic location: Exon 1, Gene Body, TSS1500, UTR5-prime, and CPGI annotations. For selection of biomarkers, we identified CPGIs with at least 8 DML having DMVs greater than 50. All loci of interest were visually inspected in Integrated Genomic Viewer (IGV).

## Additional files

Additional file 1: Figure S1. Analysis of 120 clinically annotated plasma samples from the Komen Tissue Bank, representing 40 samples from Healthy (H) individuals, 40 from disease-free survivors (DFS), and 40 from patients with metastatic breast cancer (MBC). A) Pie chart shows distribution of involved sites of distant metastases in the MBC group. B) Vertical plot shows the number of years disease free in the DFS group. Two clusters are evident. C) Plot shows cfDNA concentrations from three independent extractions obtained after samples were pooled into three groups. D) Vertical plot showing distribution of age at diagnosis for DFS and MBC patients. Age of accrual is represented for H individuals. E) Bar graph depicting the number of samples by race, for H, DFS, and MBC. (ZIP 1052 kb) Additional file 2: Table S1. Summary of clinical patient demographics. (TIFF 335 kb) Additional file 3: Figure S2. Library metrics. A) Gel image showing size distributions of template DNA for library preparation pre and post shearing. B) Bioanalyzer DNA100 electropherograms of libraries post preparation. C) Plot showing percent-alignment rates for libraries. (TIFF 718 kb) Additional file 4: Table S2. Summary of sequencing statistics. (TIFF 452 kb) Additional file 5: Figure S3. Histogram plots of the frequency of % methylation per cytosine for each sample pool by chromosome. MBC demonstrates a shift to the left compared to DFS and H for each chromosome. (TIFF 1958 kb) Additional file 6: Figure S4. Integrated Genomics Viewer screenshots of WGBS and MiSeq sequencing results shown by gene for GP5 (A), HTR1B (B), PCDH10 (C), and UNC13A (D). The top panel, separated by a thick black line, shows a histogram representing the percent methylation value for each CpG locus in each amplicon for H, DFS, and MBC. In the lower panel, histograms display the percent unmethylated reads (blue) and percent methylated reads (red) at each assayed CpG locus. Below each of these histograms are the individual reads. (ZIP 634 kb) Additional file 7: Table S3. Summary of percent methylation values for WGBS and MiSeq for 36 CpGs within GP5, HTR1B, PCDH10, and UNC13A. (TIFF 367 kb) Additional file 8: Table S4A. Ingenuity pathway analysis for 21 gene signature. Table S4B. Detailed view of 17 Genes in cancer as shown in Additional file 8: Table S4A. (TIFF 2363 kb)

## Abbreviations

cfDNA: cell-free DNA
CPGI: CpG island
DFS: disease-free survivors
DML: differentially methylated loci
DMV: differential methylation value
H: healthy individuals
IGV: Integrated Genomic Viewer
KTB: Komen Tissue Bank
MBC: metastatic breast cancer
MRD: minimal residual disease
WGBS: whole-genome bisulfite sequencing
