# Supplementary figures and images for: Whole-genome bisulfite sequencing of cell-free DNA identifies signature associated with metastatic breast cancer
Source: Clin Epigenetics. 2015 Sep 16;7(1):100. doi: 10.1186/s13148-015-0135-8 (PMC4573288; doi:10.1186/s13148-015-0135-8)

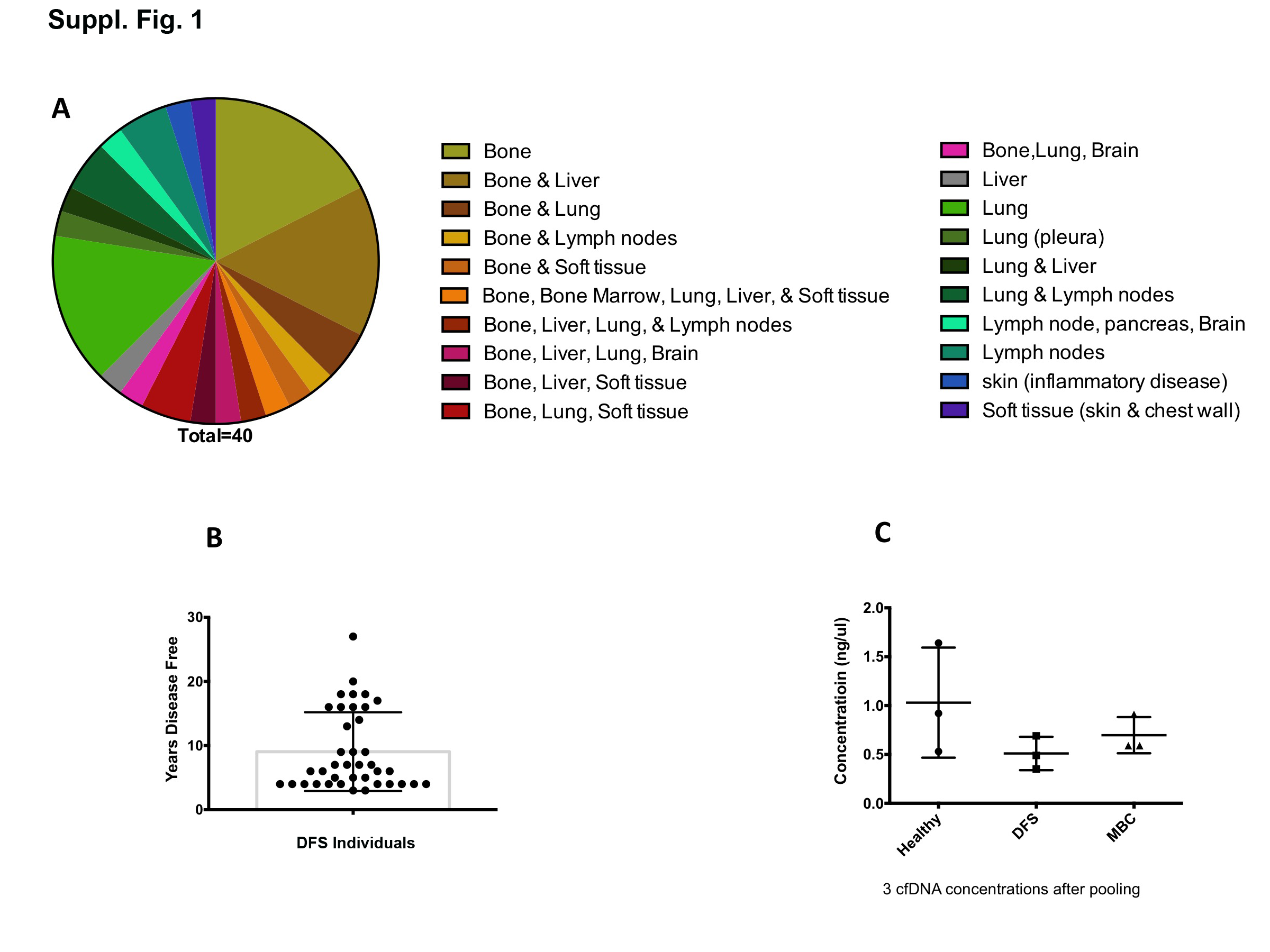

Supplement: Additional file 1: Figure S1. — Analysis of 120 clinically annotated plasma samples from the Komen Tissue Bank, representing 40 samples from Healthy (H) individuals, 40 from disease-free survivors (DFS), and 40 from patients with metastatic breast cancer (MBC). A) Pie chart shows distribution of involved sites of distant metastases in the MBC group. B) Vertical plot shows the number of years disease free in the DFS group. Two clusters are evident. C) Plot shows cfDNA concentrations from three independent extractions obtained after samples were pooled into three groups. D) Vertical plot showing distribution of age at diagnosis for DFS and MBC patients. Age of accrual is represented for H individuals. E) Bar graph depicting the number of samples by race, for H, DFS, and MBC. (ZIP 1052 kb) [file 13148_2015_135_MOESM1_ESM.zip › SupplFig1a-c_Rev_FINAL.tif]

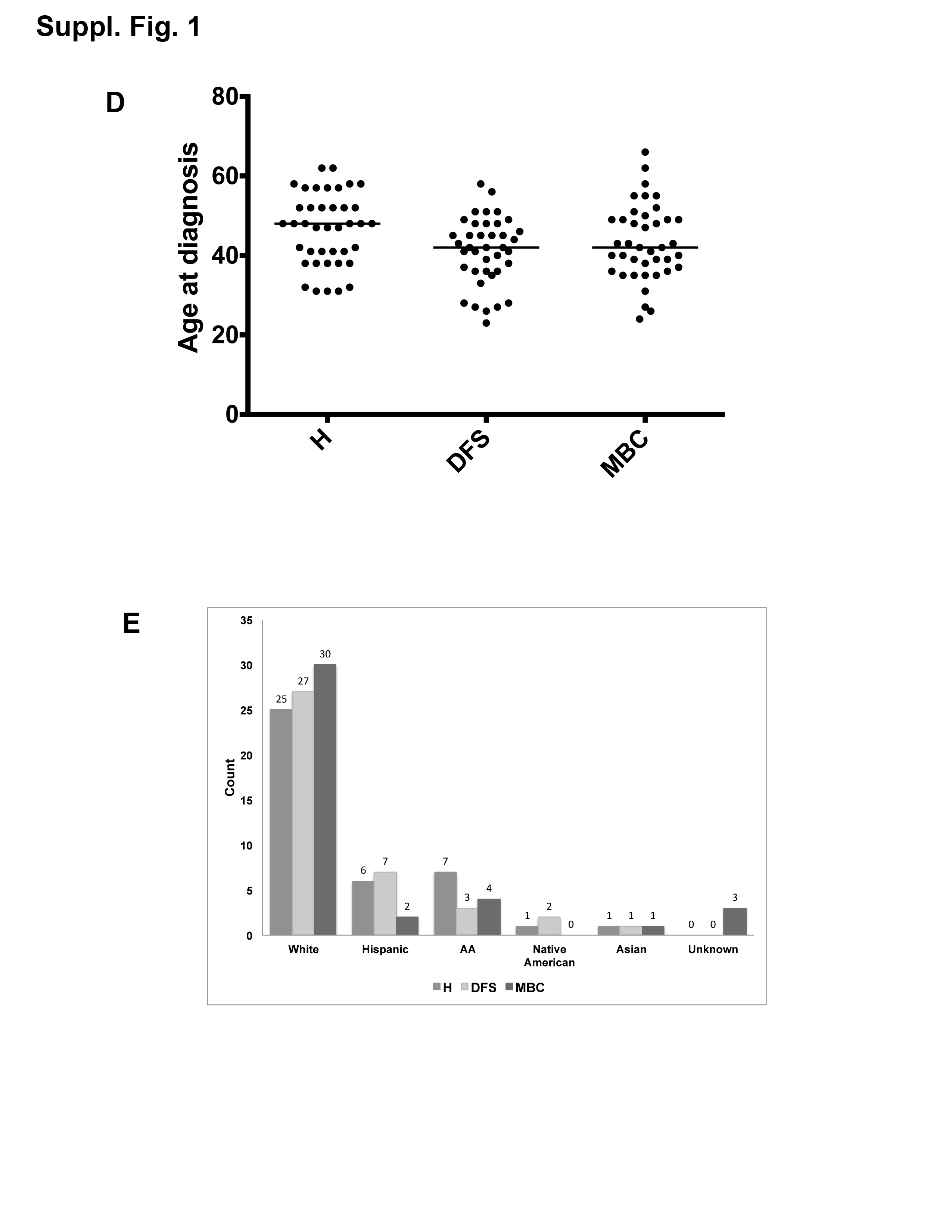

Supplement: Additional file 1: Figure S1. — Analysis of 120 clinically annotated plasma samples from the Komen Tissue Bank, representing 40 samples from Healthy (H) individuals, 40 from disease-free survivors (DFS), and 40 from patients with metastatic breast cancer (MBC). A) Pie chart shows distribution of involved sites of distant metastases in the MBC group. B) Vertical plot shows the number of years disease free in the DFS group. Two clusters are evident. C) Plot shows cfDNA concentrations from three independent extractions obtained after samples were pooled into three groups. D) Vertical plot showing distribution of age at diagnosis for DFS and MBC patients. Age of accrual is represented for H individuals. E) Bar graph depicting the number of samples by race, for H, DFS, and MBC. (ZIP 1052 kb) [file 13148_2015_135_MOESM1_ESM.zip › SupplFig1d-e_Rev_FINAL.tif]

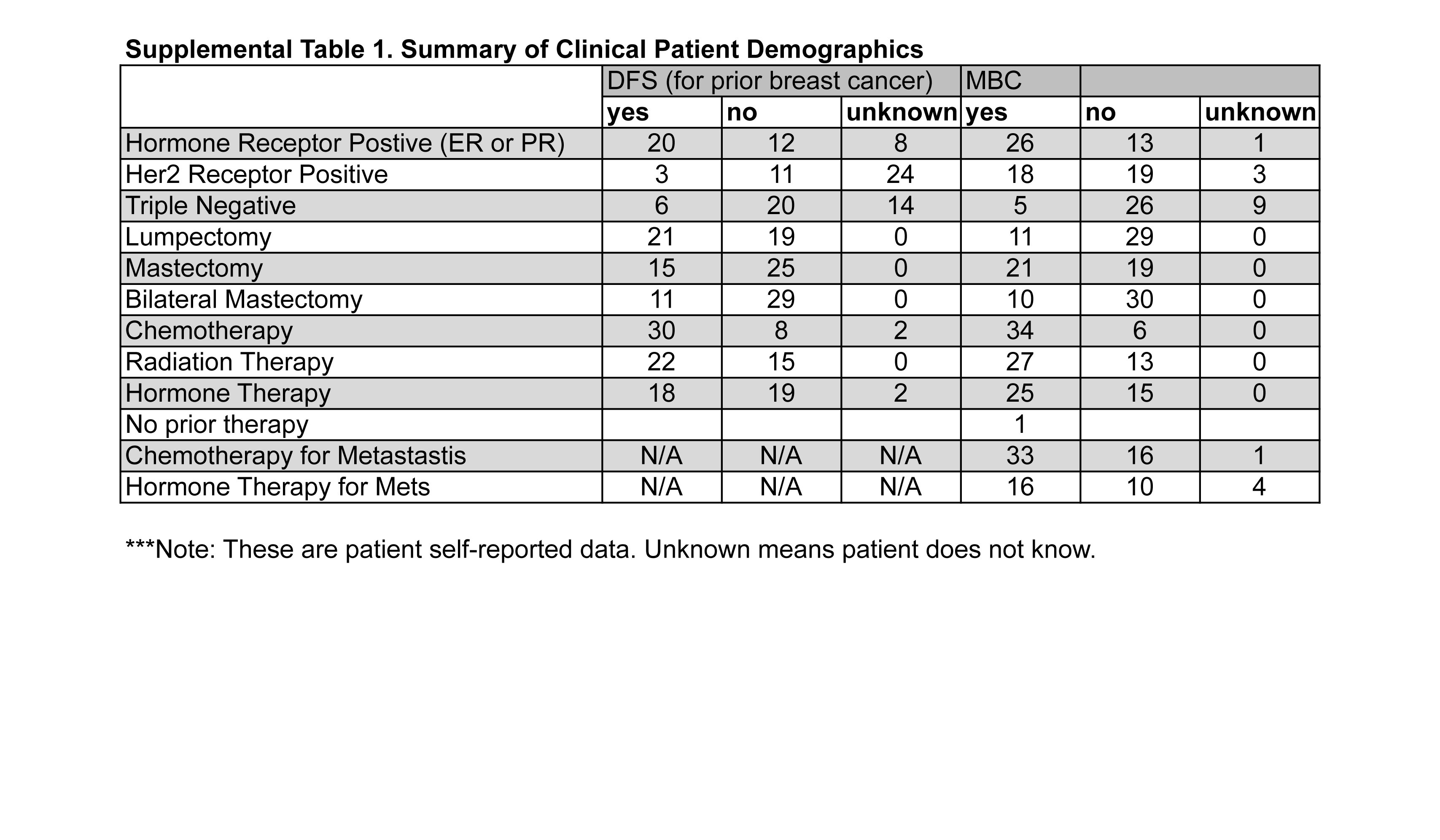

Supplement: Additional file 2: Table S1. — Summary of clinical patient demographics. (TIFF 335 kb) [file 13148_2015_135_MOESM2_ESM.tif]

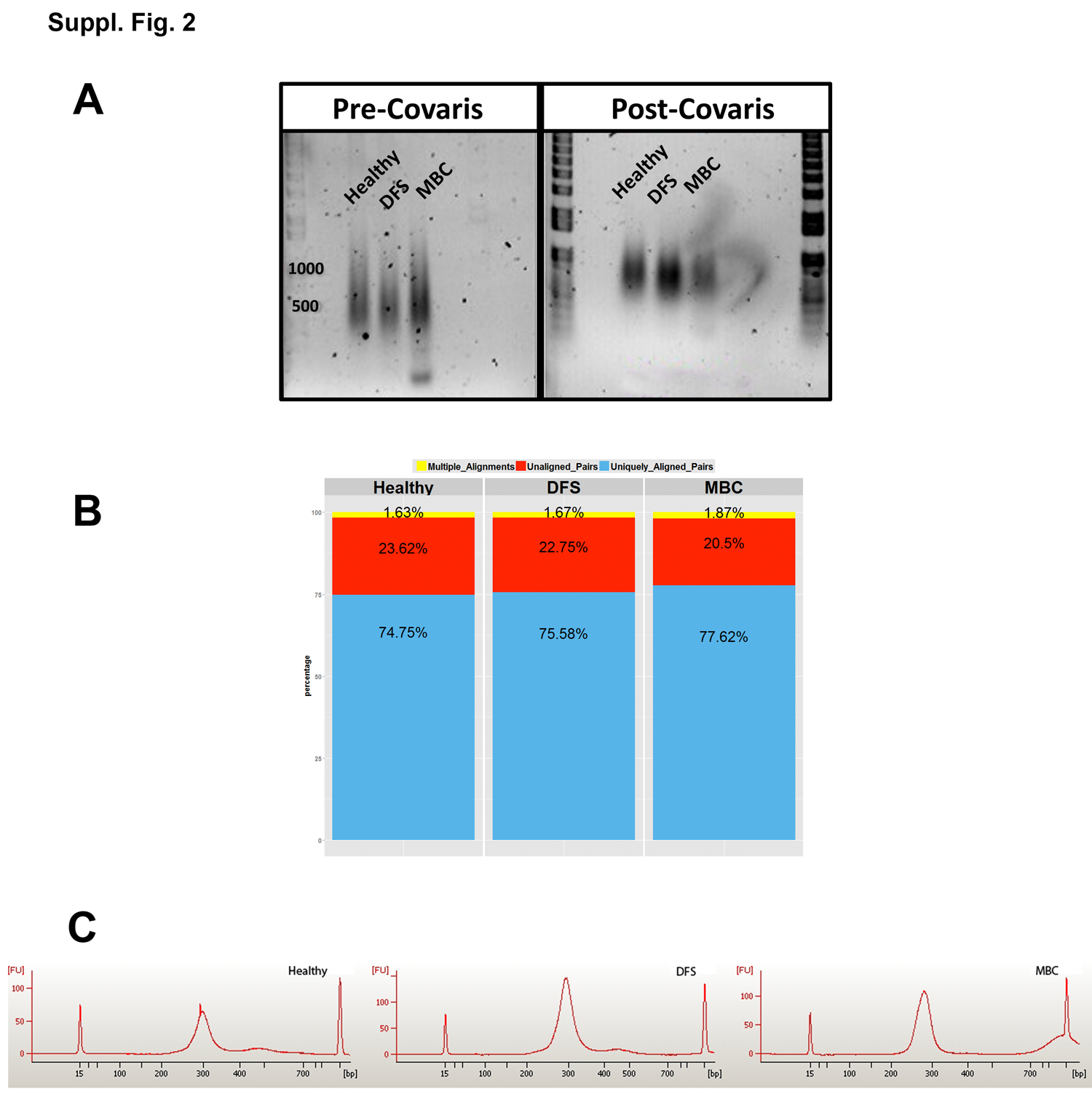

Supplement: Additional file 3: Figure S2. — Library metrics. A) Gel image showing size distributions of template DNA for library preparation pre and post shearing. B) Bioanalyzer DNA100 electropherograms of libraries post preparation. C) Plot showing percent-alignment rates for libraries. (TIFF 718 kb) [file 13148_2015_135_MOESM3_ESM.tif]

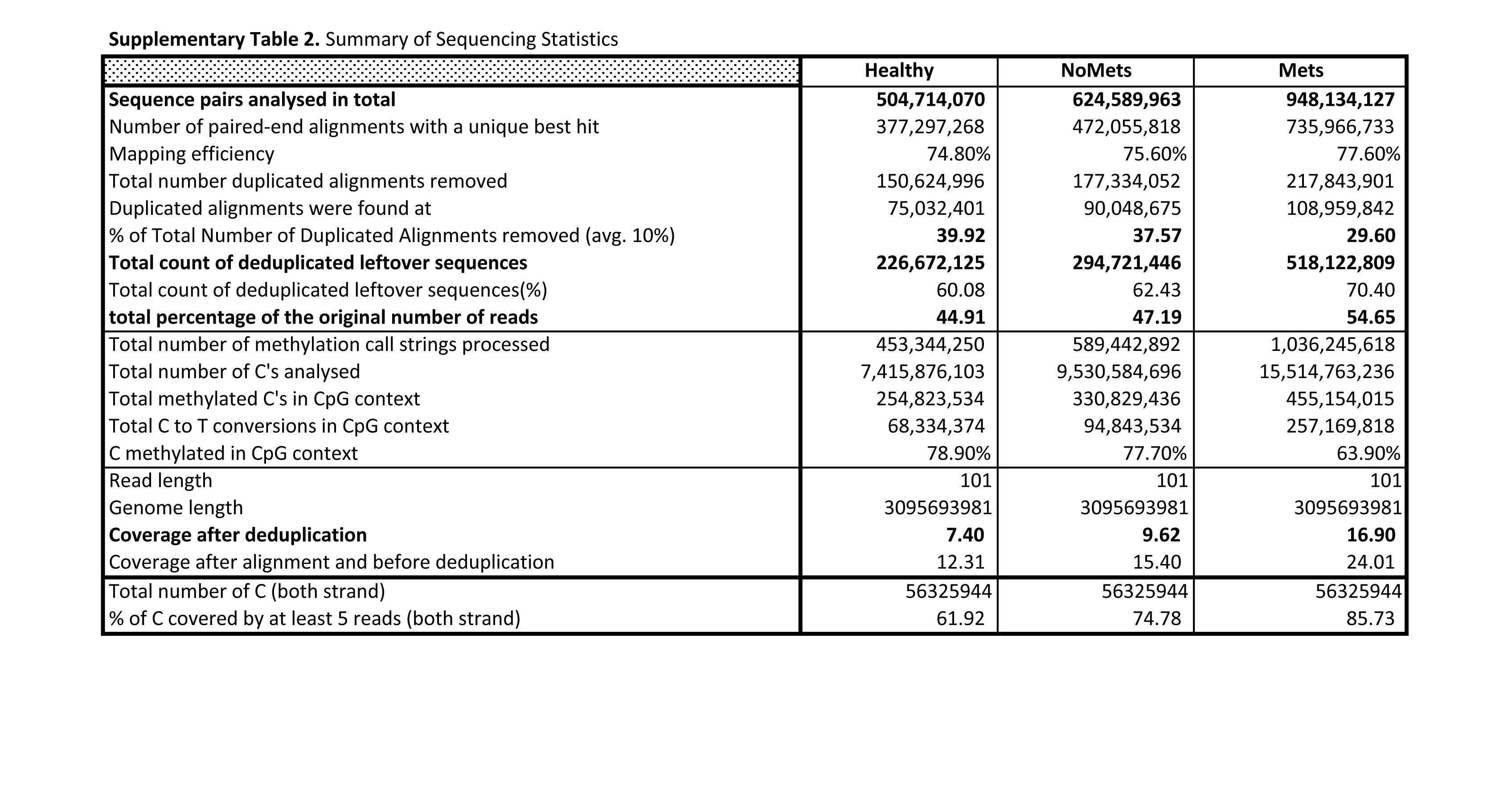

Supplement: Additional file 4: Table S2. — Summary of sequencing statistics. (TIFF 452 kb) [file 13148_2015_135_MOESM4_ESM.tif]

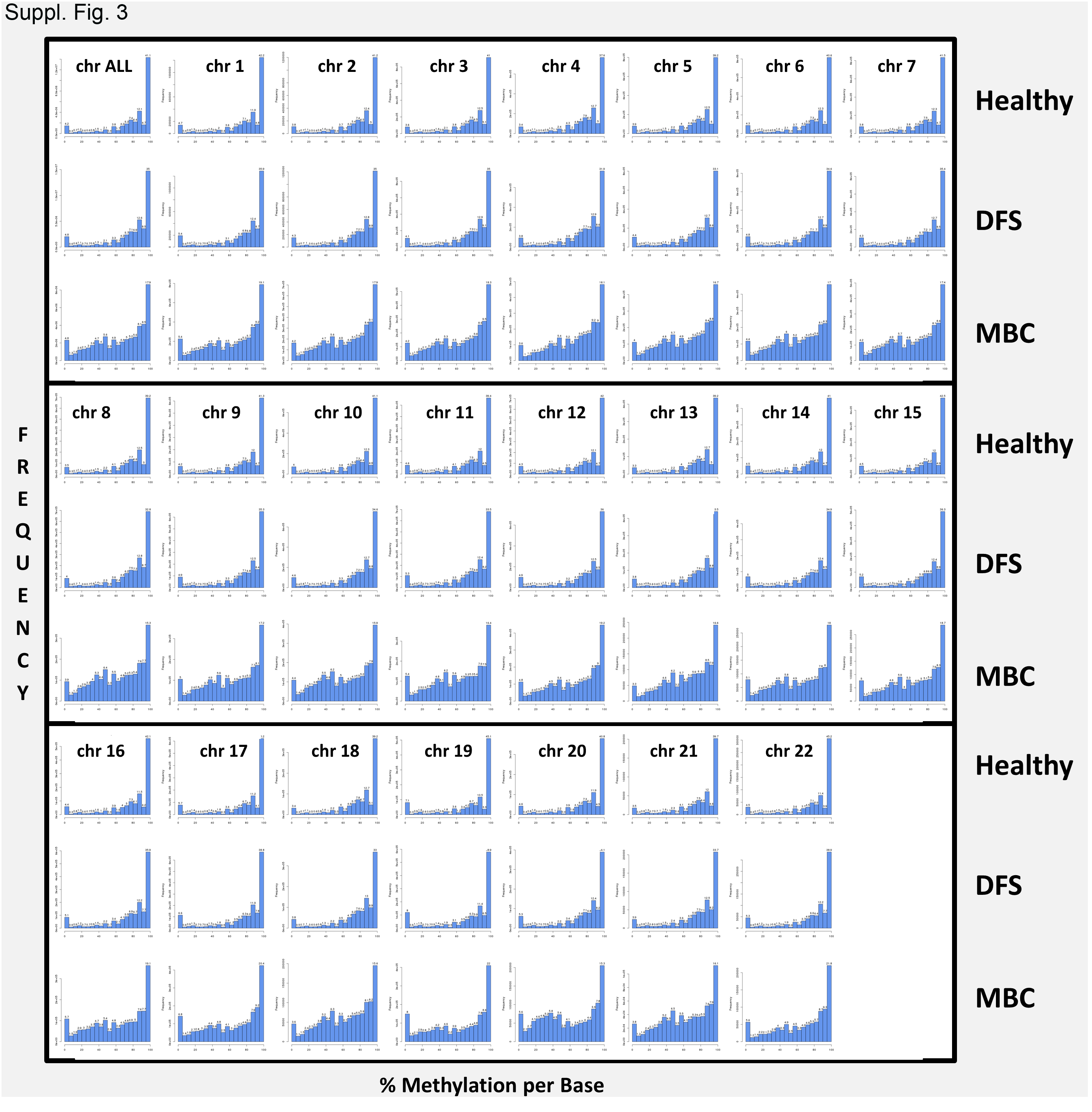

Supplement: Additional file 5: Figure S3. — Histogram plots of the frequency of % methylation per cytosine for each sample pool by chromosome. MBC demonstrates a shift to the left compared to DFS and H for each chromosome. (TIFF 1958 kb) [file 13148_2015_135_MOESM5_ESM.tif]

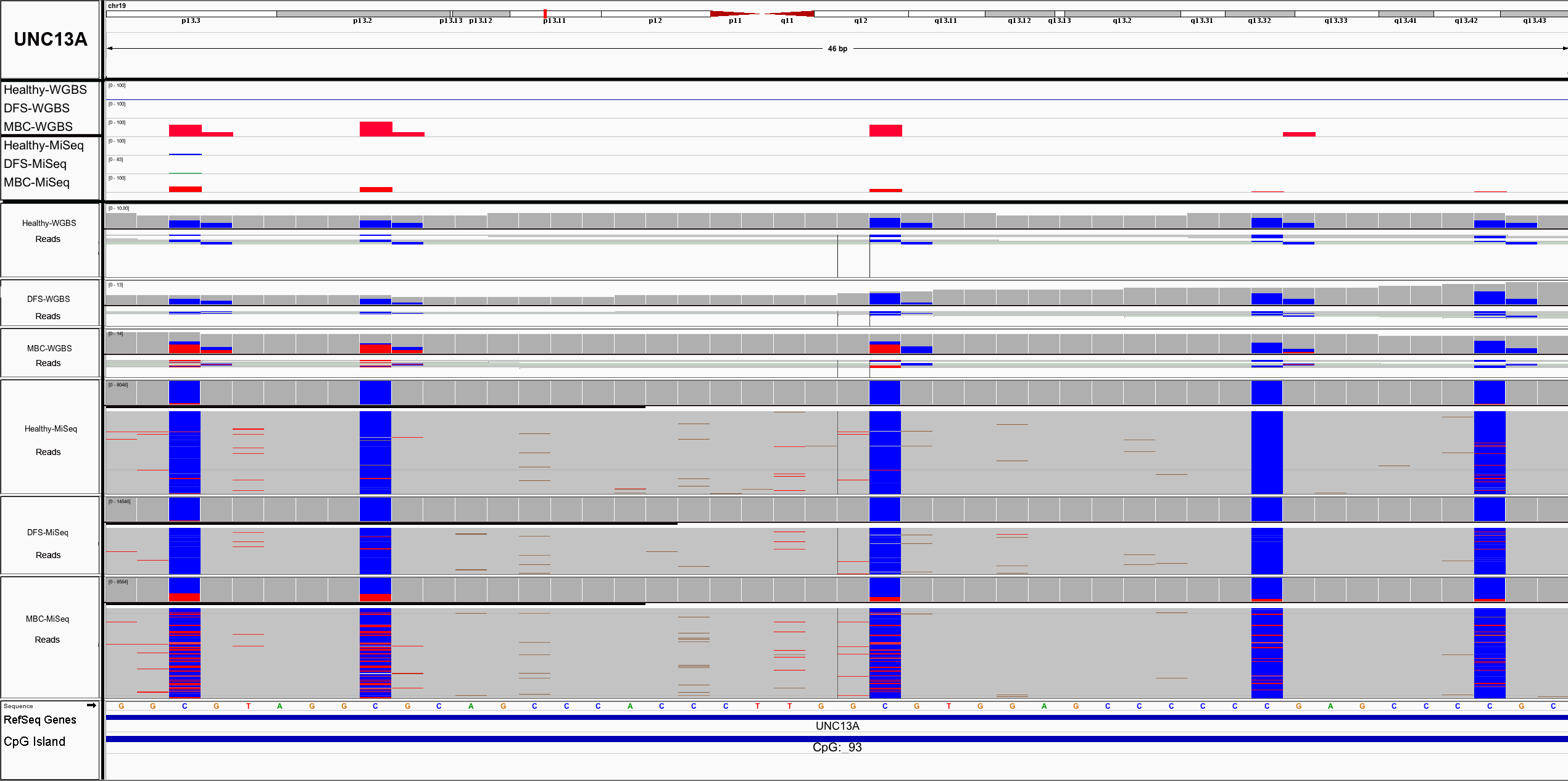

Supplement: Additional file 6: Figure S4. — Integrated Genomics Viewer screenshots of WGBS and MiSeq sequencing results shown by gene for GP5 (A), HTR1B (B), PCDH10 (C), and UNC13A (D). The top panel, separated by a thick black line, shows a histogram representing the percent methylation value for each CpG locus in each amplicon for H, DFS, and MBC. In the lower panel, histograms display the percent unmethylated reads (blue) and percent methylated reads (red) at each assayed CpG locus. Below each of these histograms are the individual reads. (ZIP 634 kb) [file 13148_2015_135_MOESM6_ESM.zip › SuppleFig4D_UNC13A_Rev_FINAL.tif]

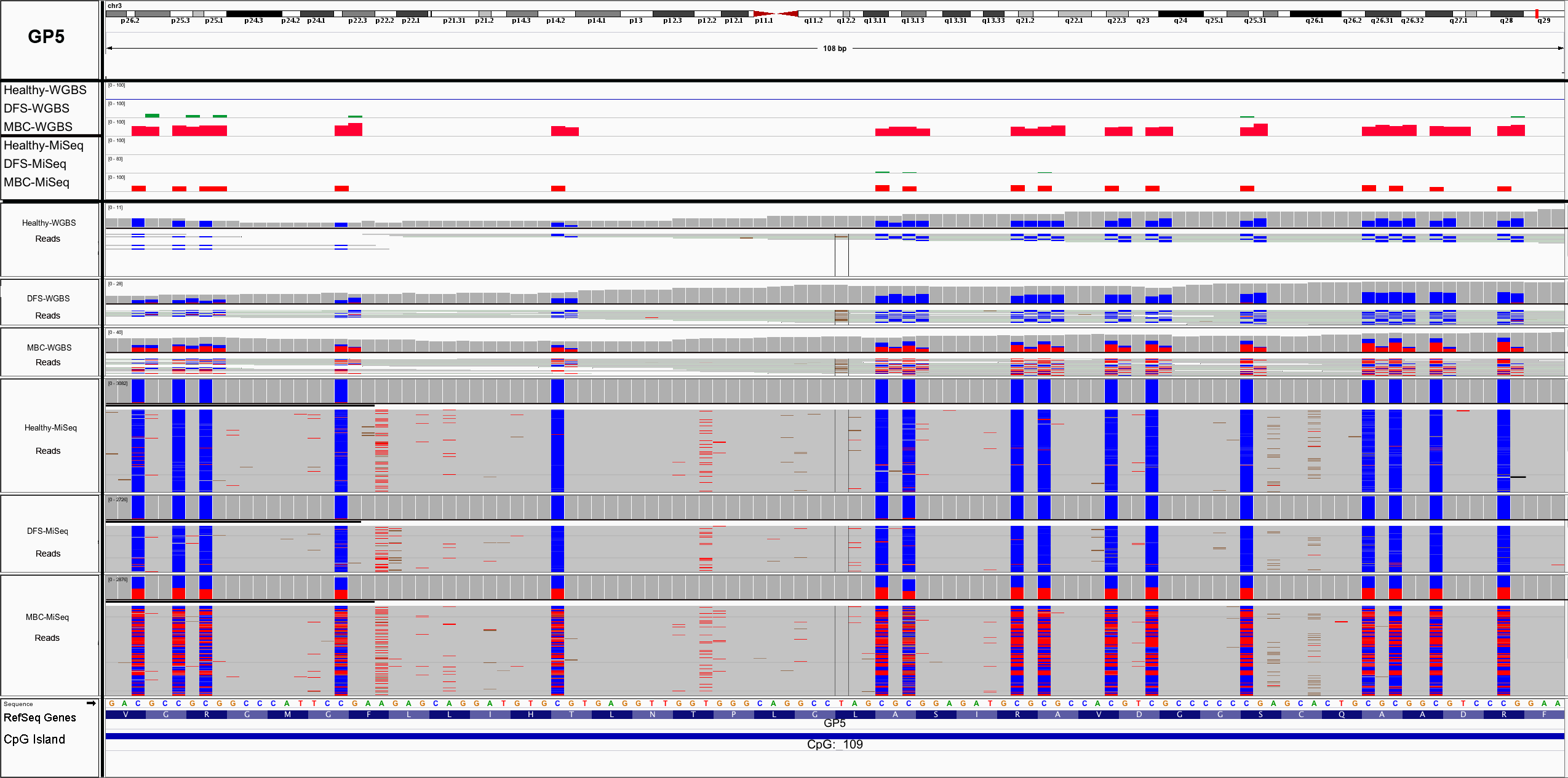

Supplement: Additional file 6: Figure S4. — Integrated Genomics Viewer screenshots of WGBS and MiSeq sequencing results shown by gene for GP5 (A), HTR1B (B), PCDH10 (C), and UNC13A (D). The top panel, separated by a thick black line, shows a histogram representing the percent methylation value for each CpG locus in each amplicon for H, DFS, and MBC. In the lower panel, histograms display the percent unmethylated reads (blue) and percent methylated reads (red) at each assayed CpG locus. Below each of these histograms are the individual reads. (ZIP 634 kb) [file 13148_2015_135_MOESM6_ESM.zip › SupplFig4A_GP5_Rev_FINAL.tif]

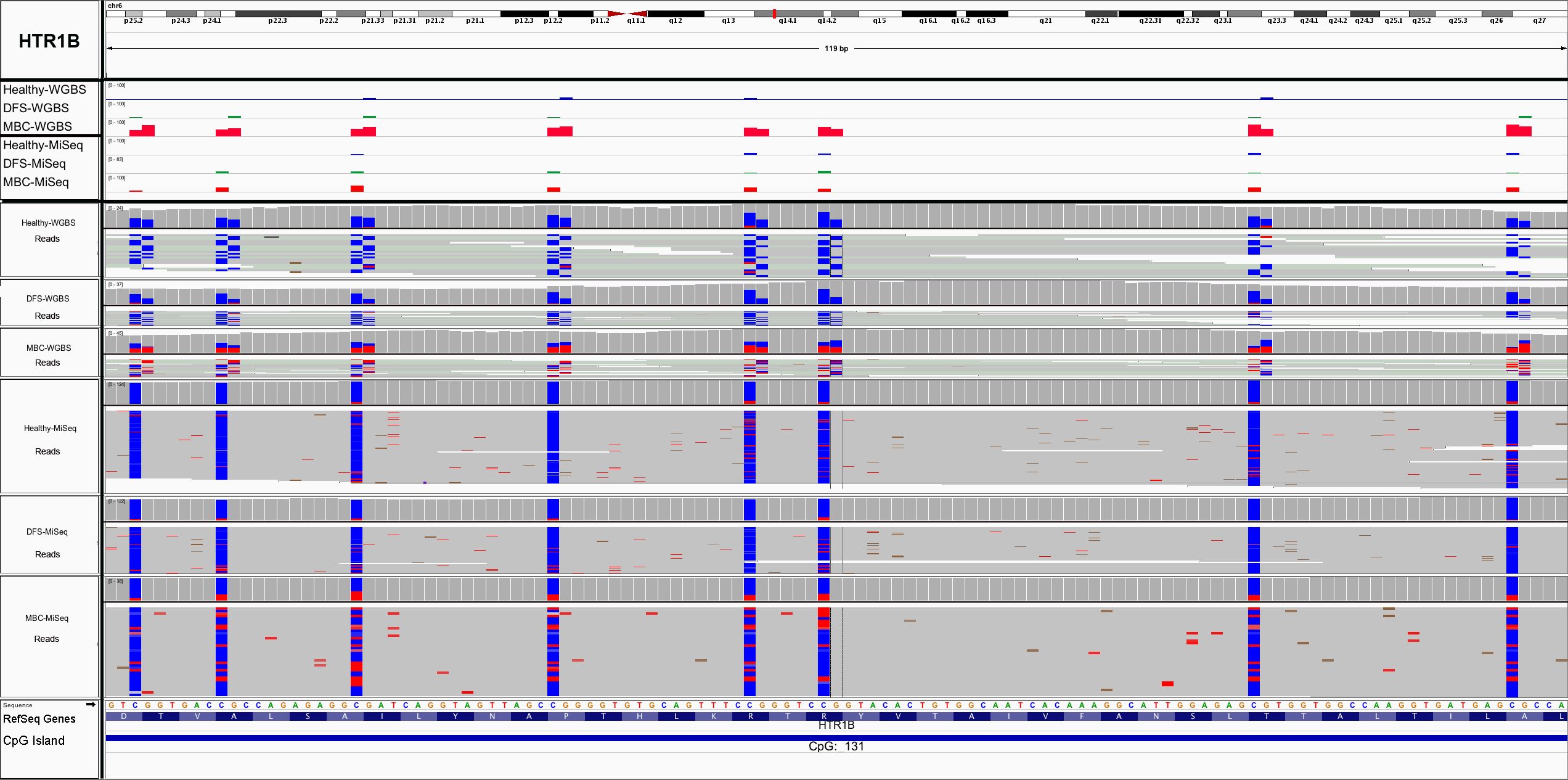

Supplement: Additional file 6: Figure S4. — Integrated Genomics Viewer screenshots of WGBS and MiSeq sequencing results shown by gene for GP5 (A), HTR1B (B), PCDH10 (C), and UNC13A (D). The top panel, separated by a thick black line, shows a histogram representing the percent methylation value for each CpG locus in each amplicon for H, DFS, and MBC. In the lower panel, histograms display the percent unmethylated reads (blue) and percent methylated reads (red) at each assayed CpG locus. Below each of these histograms are the individual reads. (ZIP 634 kb) [file 13148_2015_135_MOESM6_ESM.zip › SupplFig4B_HTR1B_Rev_FINAL.tif]

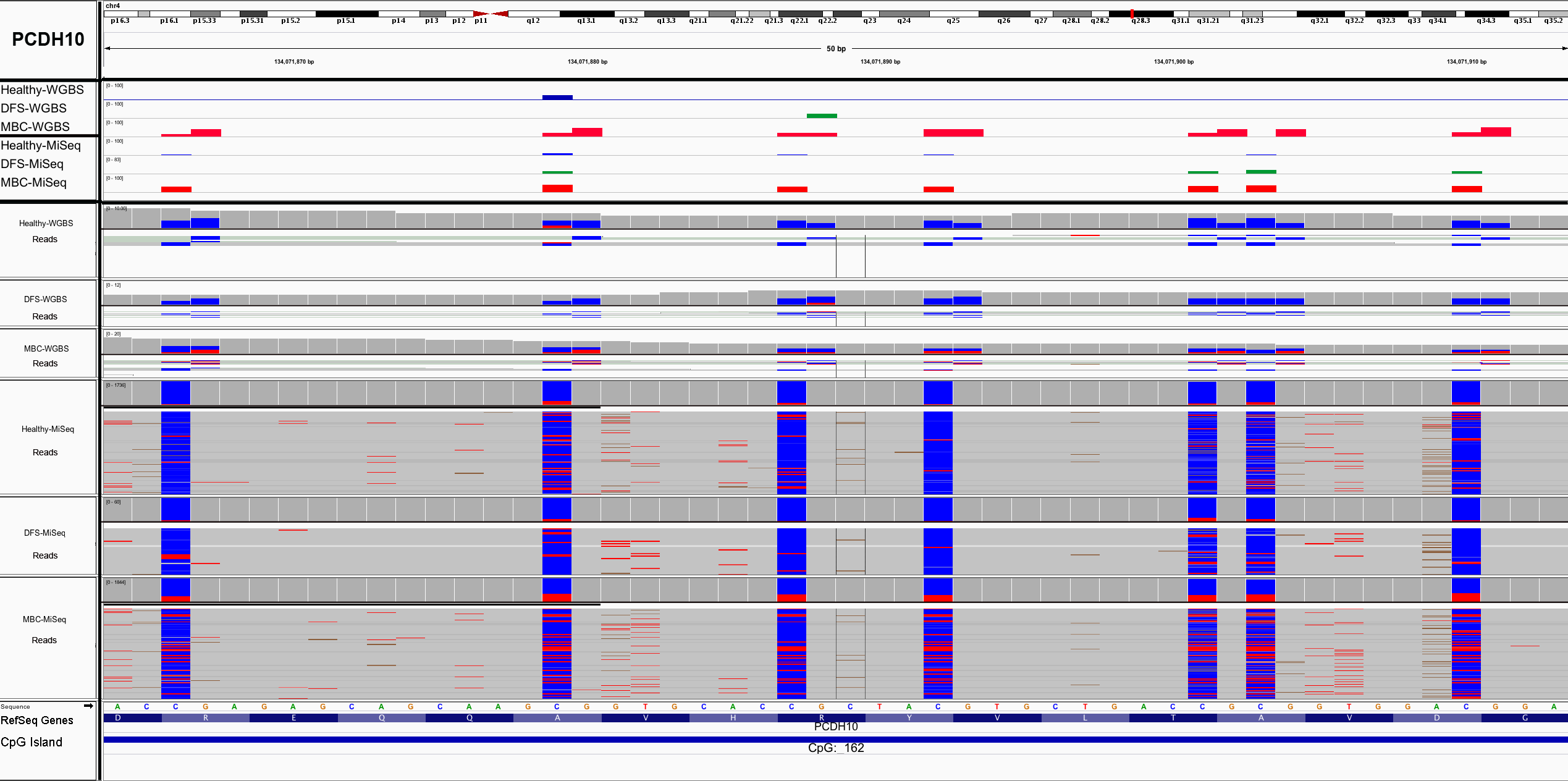

Supplement: Additional file 6: Figure S4. — Integrated Genomics Viewer screenshots of WGBS and MiSeq sequencing results shown by gene for GP5 (A), HTR1B (B), PCDH10 (C), and UNC13A (D). The top panel, separated by a thick black line, shows a histogram representing the percent methylation value for each CpG locus in each amplicon for H, DFS, and MBC. In the lower panel, histograms display the percent unmethylated reads (blue) and percent methylated reads (red) at each assayed CpG locus. Below each of these histograms are the individual reads. (ZIP 634 kb) [file 13148_2015_135_MOESM6_ESM.zip › SupplFig4C_PCDH10_Rev_FINAL.tif]

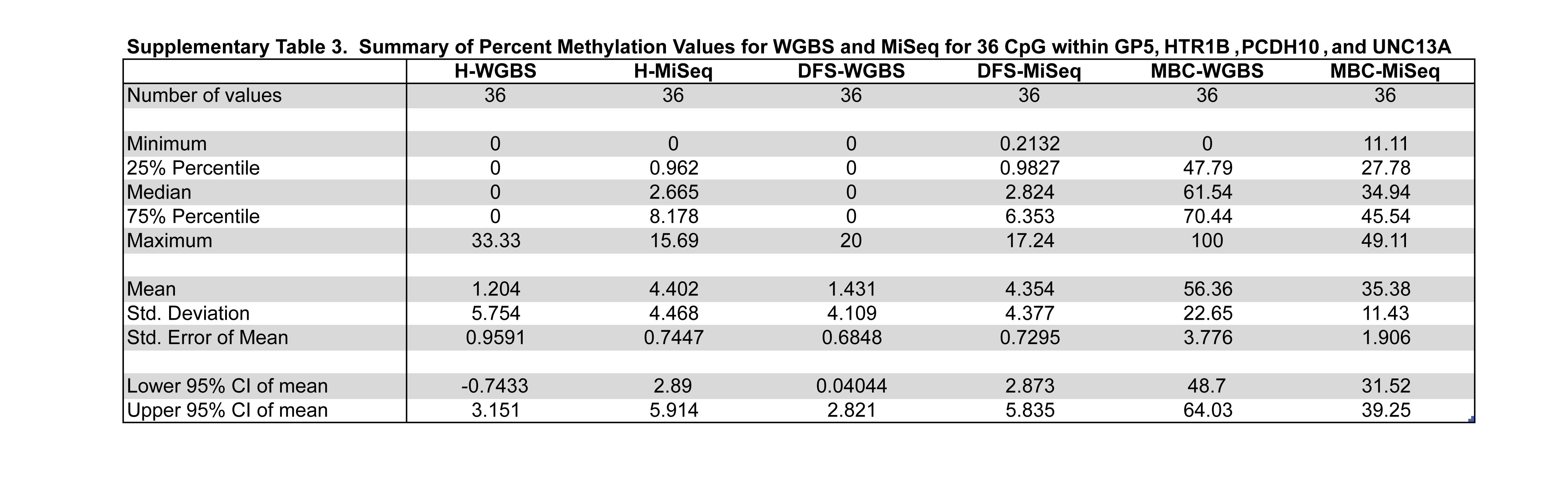

Supplement: Additional file 7: Table S3. — Summary of percent methylation values for WGBS and MiSeq for 36 CpGs within GP5, HTR1B, PCDH10, and UNC13A. (TIFF 367 kb) [file 13148_2015_135_MOESM7_ESM.tif]

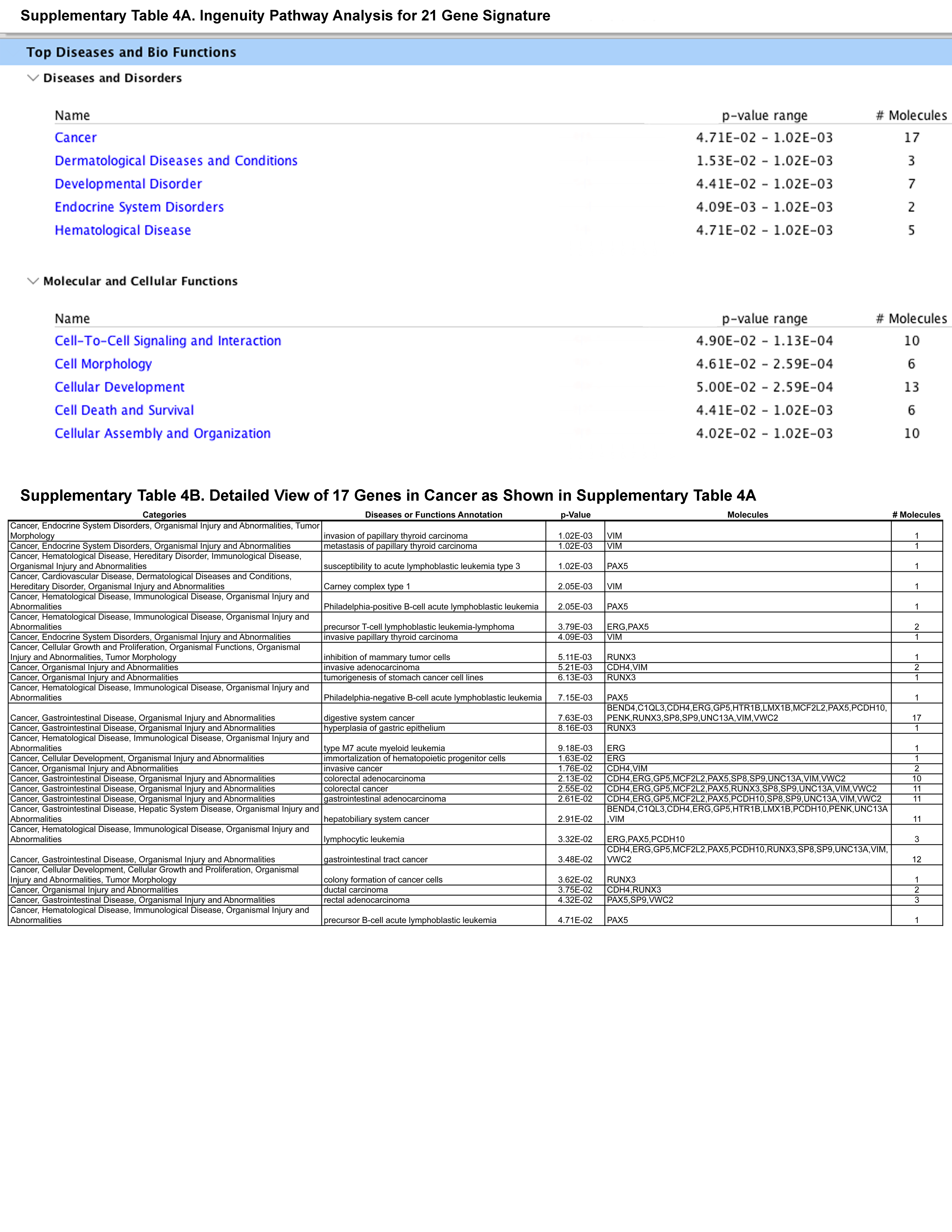

Supplement: Additional file 8: Table S4A. — Ingenuity pathway analysis for 21 gene signature. Table S4B. Detailed view of 17 Genes in cancer as shown in Additional file 8: Table S4A. (TIFF 2363 kb) [file 13148_2015_135_MOESM8_ESM.tif]
